# Effect of a Collagen-Based Compound on Morpho-Functional Properties of Cultured Human Tenocytes

**DOI:** 10.3390/cells7120246

**Published:** 2018-12-06

**Authors:** Filippo Randelli, Alessandra Menon, Alessio Giai Via, Manuel Giovanni Mazzoleni, Fabio Sciancalepore, Marco Brioschi, Nicoletta Gagliano

**Affiliations:** 1 Centro di Chirurgia dell’Anca e Traumatologia, I.R.C.C.S Policlinico San Donato, 20097 San Donato Milanese, Italy; filippo.randelli@fastwebnet.it (F.R.); alessiogiaivia@hotmail.it (A.G.V.); manuelmazzoleni4@gmail.com (M.G.M.); marco.brioschi@unimi.it (M.B.); 2 Azienda Socio Sanitaria Territoriale Centro Specialistico Ortopedico Traumatologico Gaetano Pini-CTO, 1^ Clinica Ortopedica, 20122 Milan, Italy; ale.menon@me.com (A.M.); fabio.sciancalepore@unimi.it (F.S.); 3 Department of Biomedical Sciences for Health, Università degli Studi di Milano, 20133 Milan, Italy

**Keywords:** tendinopathy, Greater Trochanter Pain Syndrome, tendon, collagen turnover, matrix metalloproteinases, cytoskeleton, focal adhesion

## Abstract

Background: Greater Trochanter Pain Syndrome (GTPS) is the main reason for recalcitrant lateral hip pain. Gluteus medius and minimus tendinopathy plays a key role in this setting. An injectable medical compound containing collagen type I (MD-Tissue, Guna) has been produced with the aim to counteract the physiological and pathological degeneration of tendons. In this study we aimed at characterizing the effect of this medical compound on cultured human gluteal tenocytes, focusing on the collagen turnover pathways, in order to understand how this medical compound could influence tendon biology and healing. Methods: Tenocytes were obtained from gluteal tendon fragments collected in eight patients without any gluteal tendon pathology undergoing total hip replacement through an anterior approach. Cell proliferation and migration were investigated by growth curves and wound healing assay, respectively. The expression of genes and proteins involved in collagen turnover were analysed by real-time PCR, Slot blot and SDS-zymography. Results: Our data show that tenocytes cultured on MD-Tissue, compared to controls, have increased proliferation rate and migration potential. MD-Tissue induced collagen type I (COL-I) secretion and mRNA levels of tissue inhibitor of matrix metalloproteinases (MMP)-1 (TIMP-1). Meanwhile, lysyl hydroxylase 2b and matrix metalloproteinases (MMP)-1 and -2, involved, respectively, in collagen maturation and degradation, were not affected. Conclusions: Considered as a whole, our results suggest that MD-Tissue could induce in tenocytes an anabolic phenotype by stimulating tenocyte proliferation and migration and COL-I synthesis, maturation, and secretion, thus favouring tendon repair. In particular, based on its effect on gluteal tenocytes, MD-Tissue could be effective in the discouraging treatment of GTPS. From now a rigorous clinical investigation is desirable to understand the real clinical potentials of this compound.

## 1. Introduction

Tendinopathy is a chronic, painful condition affecting tendons, characterized by histological modifications such as collagen fibril disorganization, increased proteoglycan and glycosaminoglycan content, and increased non-collagen extracellular matrix components, hypercellularity, and neovascularization [1]. Pain associated with tendinopathy is a very common cause of disability. Pain around the greater trochanter was in the past attributed to trochanteric bursitis, but different studies ([2,3] and references therein) have suggested that this condition is mainly determined by a tendinopathy of the gluteus medius or minimus tendons, known as Greater Trochanter Pain Syndrome (GTPS). GTPS is a clinical condition characterized by pain and tenderness at or around the greater trochanter, pain with activities such as walking and stair climbing, and lying on the affected side at night [4]. Even if it is a common condition, few effective treatments are available in the clinical practice, and are often empirical [5]. Therefore, the interest in biological and regenerative therapies aiming to improve tendon healing, such as platelet-rich plasma (PRP) or hyaluronic acid [6], has grown during the last decade. 

Treatment of tendinopathy, including Greater Trochanter Pain Syndrome and others, remains a big problem for clinicians, because its pathogenesis is still largely misunderstood, and many treatments have no real evidence. The management of GTPS is traditionally first conservative, including rest, ice-packs, non-steroidal anti-inflammatory drugs (NSAIDs), physiotherapy, and local corticosteroids injections. However, recent studies found little evidence to support physical therapy or exercise programs for gluteal tendinopathy [7]. Corticosteroid injections are widely used despite controversies regarding the use of glucocorticoid injections for the treatment of tendinopathy. A recent systematic review of literature showed that they provide short-term pain relief, but no long-term benefits [5,8]. No statistically significant differences have been reported after 1 year compared to oral NSAIDs. Some authors reported about the use of Extracorporeal Shock Wave Therapy (ESWT) with good results [9], but the evidence of most of these studies is low, and no level I studies are published in literature. Therefore, biological therapies aiming to improve tendon healing become very attractive. The only level I study on the PRP showed an improvement of pain and mHHS at 12 weeks after a single injection of PRP, compared to a single injection of corticosteroids [10]. In a recent case series, promising results have been reported with autologous tenocyte injection [11]. However, there is still little evidence to support the use of biological therapies for treatment of GTPS [12].

Tendons play key functions in musculoskeletal system in transferring forces generated by muscle contraction to the skeleton. Their mechanical properties are based on the underlying extracellular matrix (ECM) structure and composition, mainly consisting of type I collagen (COL-I) [13,14,15]. Tenocytes are tendon specialized fibroblasts interspersed between collagen fibers, responsible for the metabolic activity and structure of tendon. They are involved in collagen turnover pathways and act as mechanosensors playing key roles in modifying gene expression for ECM components in response to mechanical forces acting on tendons [14,16,17].

The aim of this study was to investigate in vitro the effect of MD-Tissue^®^, an injectable collagen-based medical compound with therapeutic potential (registered as a medical device in different countries) containing swine collagen as main component, on human tenocytes, with particular attention on collagen turnover pathways, in order to understand the molecular mechanisms triggered by this medical compound and, therefore, how it could act in favoring tendon homeostasis and repair. We were particularly interested in clinical situations such as GTPS; for this purpose we analyzed gluteal tenocytes.

The production process of MD-Tissue by the manufacturer allows to obtain a pure product with a standardized molecular weight, having the chemical-physical characteristics to guarantee safety in clinical use. No side effects, allergic reactions, nor drug interactions have been observed. Clinical data reported that treatment with MD-Knee, a collagen-based medical compound similar to MD-Tissue, for up to 6 months was generally well tolerated, and no systemic adverse events or septic complications were observed [18,19]. 

The characterization in vitro of the molecular mechanisms triggered by MD-Tissue will be pivotal to plan specific clinical trials. 

## 2. Materials and Methods

All subjects gave their informed consent for inclusion before they participated in the study. The study was conducted in accordance with the Declaration of Helsinki, and the protocol was approved by the Local Ethics Committee (San Raffaele Hospital Ethical Committee, Milan, Italy) of the coordinating Institution (IRCCS Policlinico San Donato, Milan, Italy) (63/INT/2017) Inclusion and exclusion criteria are listed in Table 1.

### 2.1. Samples

Fragments from human gluteal tendon were obtained from 8 patients (mean age 64.8 ± 7.2 years, 4 males and 4 females) without any gluteal tendon pathology and undergoing total hip replacement through an anterior approach. For each sample we analyzed the mid-substance of the collected tendon, representing the region with the typical structure of the dense regular connective tissue. 

### 2.2. Cell Cultures

Tendon fragments were immediately washed in sterile PBS and plated in T25 flasks, incubated in Dulbecco’s Modified Eagle Medium (DMEM) supplemented with 10% heat-inactivated fetal bovine serum (FBS), antibiotics (100 U/mL penicillin, 0.1 mg/mL streptomycin), and ascorbic acid (200 µM) at 37 °C in a humidified atmosphere containing 5% CO_2_. When tenocytes grew out from the explant, they were trypsinized (0.025% trypsin-0.02% EDTA) for secondary cultures and plated in T75 flasks. Viability was assessed by the Trypan blue exclusion method. For evaluations, confluent human tenocytes were used between the fourth and fifth passage. For SDS-zymography cells were cultured in serum-free DMEM. Tenocytes derived from each patients and cell supernatants were prepared in duplicate and were analyzed after 24, 48, and 72 h.

### 2.3. Coating with MD-Tissue

MD-Tissue^®^, kindly provided by Guna (Milan, Italy), is an injectable medical compound based on swine collagen (100 µg/2 mL ampoules). It contains ascorbic acid, magnesium gluconate, pyridoxin hydrochloride, riboflavin, thiamine hydrochloride, NaCl and water as excipients. Swine collagen is similar to human collagen and due to the high biocompatibility with humans and very low risk of adverse effects, it has been used in various clinical fields. MD-Tissue is an injectable compound but we hypothesized that it could act as a mechanical scaffold to influence tenocyte metabolism. Thus, MD-Tissue was used as a substrate for cell cultures. However, as previously demonstrated [20], some of the collagen used for the coating in cell cultures comes out contributing to the mechanical stimulation of tenocytes. 

To obtain a thin coating, MD-Tissue (50 µg/mL) was added to multi-wells or T25 flasks (3 mL per T25 flask and 6-well multi-well plates, ad 500 µL per 24-well multi-well plates). After an incubation of at least 3–4 h at room temperature to ensure that collagen has adhered to the plastic, excess fluid was removed from the coated surface that was dried for at least 2 h under the laminar flux hood. Coated plastic was used immediately or stored at 4 °C. 

Cells cultured on uncoated cell culture plastic (NC) were used as untreated controls.

### 2.4. Growth Curves

Cell growth was assessed by growth curves. Tenocytes were plated in triplicate samples in 6-well multi-well plates at the same cell density (100,000 cells/well). Cell number was determined using a Neubauer chamber after 24, 48, and 72 h in tenocytes in the proliferative phase.

### 2.5. Immunofluorescence Analysis

For fluorescence microscopy, tenocytes were cultured on uncoated (NC) or MD-Tissue coated 12-mm diameter round coverslips put into 24-well culture plates, as previously described [21]. Briefly, for actin cytoskeleton analysis, cells were incubated with 50 µM rhodamine-phalloidin (Sigma-Aldrich, St. Louis, MO, USA) and for vinculin detection, cells were incubated for 1 h at room temperature with the rabbit polyclonal antibody anti-vinculin (1:600 in PBS, Sigma-Aldrich, St. Louis, MO, USA). The secondary antibody was an anti-rabbit/Alexa488 (1:500, Life Technologies, Carlsbad, CA, USA). Cells were photographed by a digital camera connected to a Nikon Eclipse 80i microscope.

### 2.6. Real-Time PCR

Gene expression was analyzed by real-time RT-PCR as previously reported in samples run in triplicate [22]. GAPDH was used as endogenous control to normalize the differences in the amount of total RNA in each sample. The primers sequences were the following: Glyceraldehyde 3-phosphate dehydrogenase (GAPDH): sense CCCTTCATTGACCTCAACTACATG, antisense TGGGATTTCCATTGATGACAAGC; Long lysyl hydroxylase 2 (LH2b): sense CCGGAAACATTCCAAATGCTCAG, antisense GCCAGAGGTCATTGTTATAATGGG; Tissue inhibitor of matrix metalloproteinase 1 (TIMP-1): sense GGCTTCTGGCATCCTGTTGTTG, antisense AAGGTGGTCTGGTTGACTTCTGG; vinculin: sense GGAGGTGATTAACCAGCCAAT, antisense AATGATGTCATTGCCCTTGC; Focal adhesion kinase (FAK): sense GTCTGCCTTCGCTTCACG, antisense GAATTTGTAACTGGAAGATGCAAG; Nanog sense CCCCAGCCTTTACTCTTCCTA, antisense CCAGGTTGAATTGTTCCAGGTC. Each sample was analyzed in triplicate in a Bioer LineGene 9600 thermal cycler (Bioer, hangzhou, China) after 40 cycles. The cycle threshold (Ct) was determined and gene expression levels relative to that of GAPDH were calculated. To confirm the reliability of gene expression results, mRNA levels of target genes were normalized on a second housekeeping gene, the 18s ribosomal RNA. The expression pattern obtained was similar (data not shown).

### 2.7. Slot Blot

Collagen type I and III (COL-I, COL-III), matrix metalloproteinase (MMP)-1 protein levels secreted by tenocytes were assessed in duplicate samples by Slot blot in serum free cell culture medium, as previously detailed [21]. Membranes were incubated for 1 h at room temperature in monoclonal antibody to COL-I (1:1000 in TBST) (Sigma-Aldrich, Milan, Italy), COL-III (1:2000 in TBST) (Sigma-Aldrich, Milan, Italy), MMP-1 (1 µg/mL in TBST) (Millipore, Milan, Italy). Immunoreactive bands, revealed by the Amplified Opti-4CN substrate (Amplified Opti-4CN, Bio Rad, Italy), were scanned densitometrically (UVBand, Eppendorf, Italy). 

### 2.8. SDS-Zymography

SDS-zymography was used to analyze MMP-2 activity of secreted protein in cell culture medium, as previously described [21]. MMP gelatinolytic activity, detected after staining the gels with Coomassie brilliant blue R250 as clear bands on a blue background, were quantified by densitometric scanning (UVBand, Eppendorf, Italy).

### 2.9. Wound Healing Assay

Cell migration in adult and aging tenocytes was analyzed by wound healing assay in uncoated (NC) and MD-Tissue coated 6-wells multi-well plates [22]. The “scratch” was created in confluent tenocytes using a p 200 pipet tip. After cell debris removal by DMEM washing, multi-well plates were incubated at 37 °C and observed under an inverted microscope at different time points. Digital images were captured by a digital camera after 0 and 24 h, and the size of the “scratch” was measured to obtain the migration potential. 

### 2.10. Statistical Analysis

Statistical analysis was performed using GraphPad Prism v 6.0 software (GraphPad Software Inc., San Diego, CA 92108, USA). Data were obtained from two replicate experiments for each of the patients-derived cell lines cultured in duplicate and were expressed as mean ± standard deviation (SD). Comparison of groups was calculated using independent samples two-tailed t test. Differences associated with p-values lower than 5%, at a confidence level of 95%, were to be considered significant.

## 3. Results

### 3.1. Cell Growth

**Figure 1:**
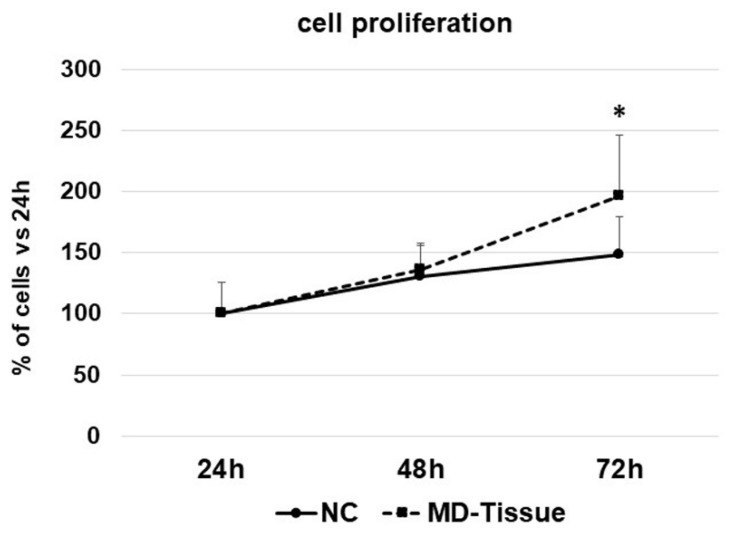
Growth curves of tenocytes grown without (NC) or on MD-Tissue at the indicated time points. Data are expressed as percentages vs. the time point T24 and are mean + SD. * *p* < 0.05 vs. 72 h NC.

Cell proliferation evaluated by growth curves showed that tenocytes cultured on uncoated (NC) or MD-Tissue coated 6-well multi-well plates had the same proliferation rate after 24 and 48 h. By contrast, cell proliferation significantly increased in tenocytes grown on MD-Tissue after 72 h (*p* < 0.05 vs. 72 h NC) (Figure 1). 

### 3.2. Expression of Genes and Proteins Related to Collagen Turnover

**Figure 2:**
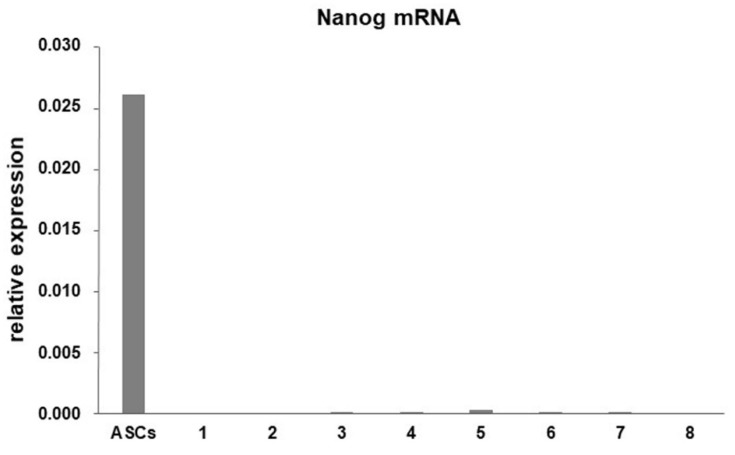
Bar graphs showing mRNA levels for Nanog in adipose tissue-derived stem cells (ASCs) and the eight cell cultures of tenocytes assessed by real-time PCR. Data were normalized on Glyceraldehyde 3-phosphate dehydrogenase (GAPDH) gene expression.

Since it was demonstrated that, using different methods to isolate tenocytes from tendon fragments, the obtained cell population can be a mixed population of terminally differentiated tenocytes and progenitor cells [23], we first characterized our cells determining the expression of Nanog, one of the main mesenchymal stem cell markers [24]. Adipose tissue-derived stem cells (ASCs) (kindly provided by Dr. Anna Brini, University of Milan) were used as positive control. We found that Nanog is almost undetectable in all the considered cell cultures obtained from tendon fragments, while it is highly expressed in ASCs (Figure 2), showing that cells used in this study are mostly differentiated tenocytes. 

**Figure 3:**
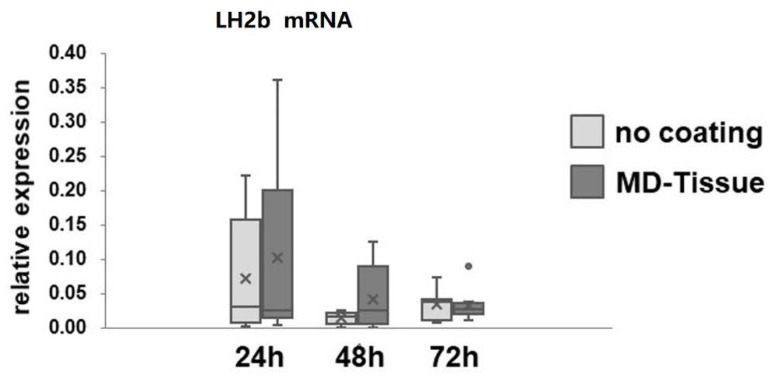
Bar graphs showing mRNA levels for Long lysyl hydroxylase 2 (LH2b) in NC and MD-Tissue tenocytes (COL) assessed by real-time PCR. Data were normalized on GAPDH gene expression and are expressed as mean ± SD for at least two independent experiments for the eight samples run in duplicate. ×: mean; •: outlier sample.

**Figure 4:**
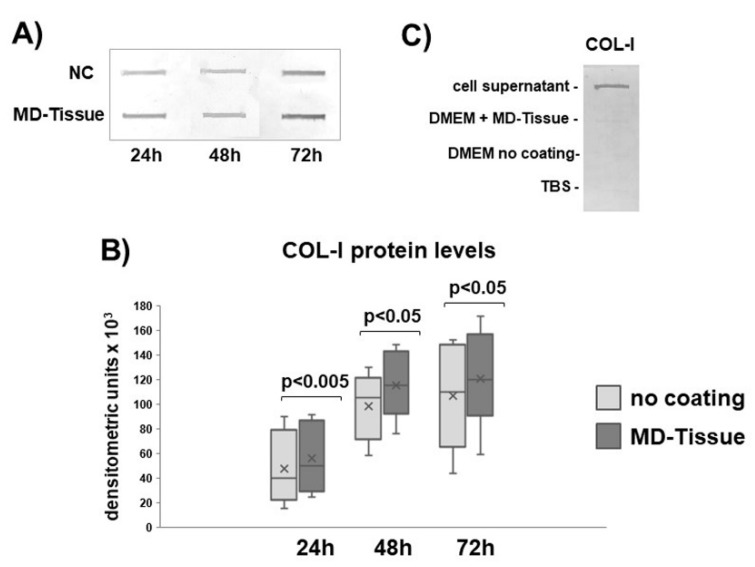
Representative slot blot analysis for collagen type I (COL-I) expression (**A**) expression in cell-culture medium of tenocytes cultured without coating (NC) or on MD-Tissue. Bar graphs displaying COL-I (**B**) protein levels analyzed densitometric scanning of immunoreactive bands in panel A. Data are expressed as mean ± SD for the 8 samples. (**C**) Slot blot analysis for COL-I showing that COL-I expression originates from tenocytes. ×: mean.

Collagen maturation was assessed by analyzing LH2b gene expression by real time PCR, involved in cross-linking of newly synthetized collagen. LH2b mRNA levels were unaffected by MD-Tissue at all the considered time points (Figure 3). Slot blot analysis revealed that COL-I protein levels secreted in cell culture medium by tenocytes cultured on MD-Tissue were increased (+25%, +19% and +18%, respectively, for COL vs. NC after 24, 48 and 72 h). This increase was statistically significant in cells cultured on COL, compared to NC, at all the considered time points (*p* < 0.005, *p* < 0.05 and *p* < 0.05 for MD-Tissue vs. CT, respectively, at 24, 48 and 72 h) **(**Figure 4A,B). To demonstrate that collagen expression detected by Slot blot originates from tenocytes and not from the coating, we prepared a Slot blot to analyze COL-I expression in the same volume (100 µL) of tenocyte culture medium, DMEM from a MD-Tissue coated well, DMEM only and TBS buffer. The result shows that an immunoreactive band is detected only for tenocytes, demonstrating that collagen originates only from cells and not from the coating (Figure 3C). By contrast, COL-III protein levels were similarly secreted by NC and MD-Tissue tenocytes (data not shown).

**Figure 5:**
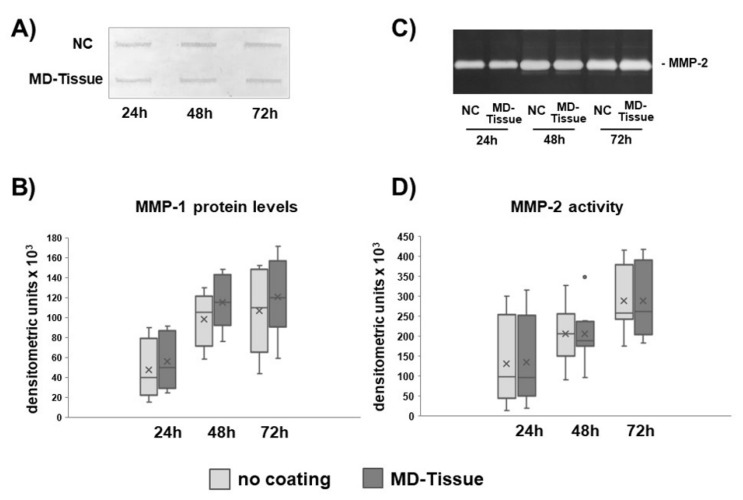
Representative slot blot for matrix metalloproteinase-1 (MMP-1) levels (**A**) and representative SDS-zymography showing MMP-2 activity in serum-free cell supernatants of NC and MD-Tissue tenocytes. Bar graphs showing MMP-1 protein levels (**C**) and MMP-2 activity (**D**) after densitometric analysis of immunoreactive and lytic bands, respectively. Data obtained from the eight samples are expressed as a % of densitometric units vs NC and are ± SD. NC: no coating. ×: mean; •: outlier sample.

**Figure 6:**
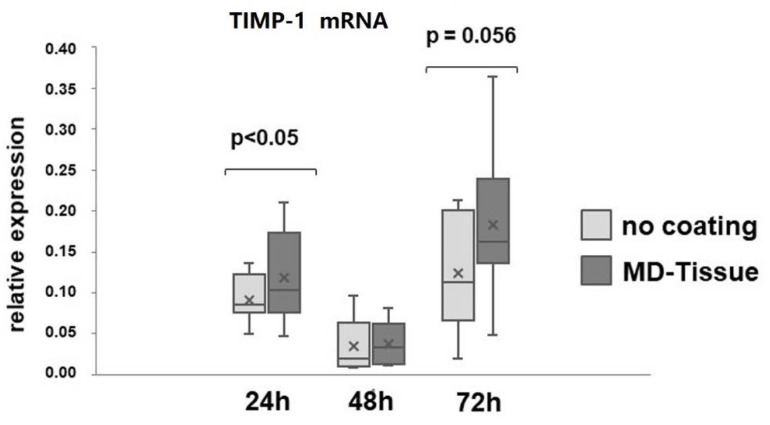
Bar graphs showing tissue inhibitor of matrix metalloproteinase-1 (TIMP-1) gene expression after normalization on GAPDH mRNA levels. Data obtained from the eight samples are expressed as mean ± SD. ×: mean.

Collagen degradation analysis revealed that MMP-1 levels (Figure 5A,B), and MMP-2 activity (Figure 5C,D) were similar in tenocytes cultured without coating (NC) or on MD-Tissue. By contrast, gene expression for TIMP-1, the main inhibitor of MMP-1, was strongly affected by MD-Tissue. In fact, gene expression was significantly up-regulated after 24 h (*p* < 0.05) and tended to a significant up-regulation after 72 h (*p* = 0.056) (Figure 6). Some interindividual differences in MMPs expression and TIMP-1 mRNA levels were observed, showing unchanged, increased or decreased levels. However, a strong correlation (*p* = 0.056) was observed between MMP-1 protein levels and TIMP-1 gene expression for tenocytes cultured on MD-Tissue at 72 h.

### 3.3. Cytoskeleton Arrangement and Focal Adhesion

**Figure 7:**
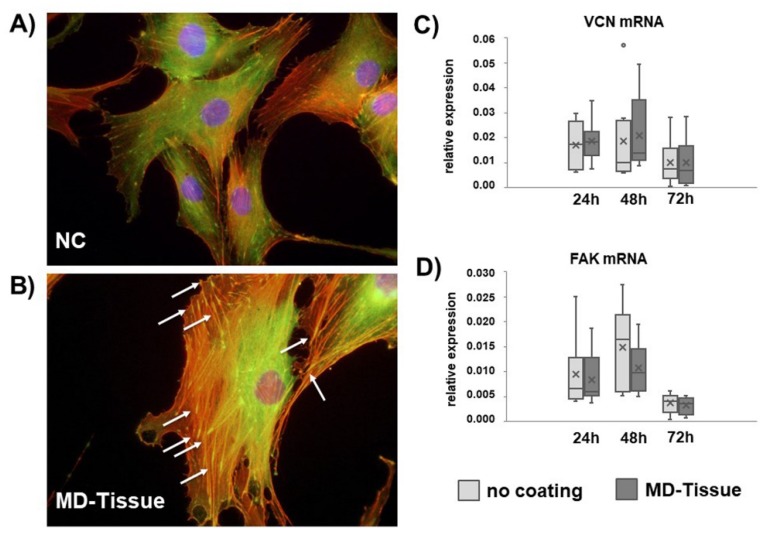
Immunofluorescence analysis for vinculin (green) in control tenocytes (NC) (**A**) and tenocytes cultured on MD-Tissue (**B**). Actin filaments are stained using rhodamine-phalloidin labeling. Nuclei are stained in blue by DAPI. Original magnification: 60×. Bar graphs showing VCN (**C**) and FAK (**D**) gene expression relative to GAPDH mRNA levels in NC and MD-Tissue tenocytes (COL). Data obtained from the eight samples are expressed as mean ± SD. ×: mean; •: outlier sample.

Fluorescent microscopy analysis for F-actin (Figure 7A,B) revealed that actin filaments seem unaffected by MD-Tissue. In both experimental conditions, they are long and mostly longitudinally oriented in the cytoplasm.

In order to investigate whether MD-Tissue influences the ability of tenocytes to form focal adhesions needed for cell migration, we analyzed the gene expression for vinculin (VCN) and Focal adhesion kinase (FAK), key proteins involved in the formation of the adhesion plaque. VCN and FAK mRNA levels, although with some interindividual differences, were unaffected by MD-Tissue (Figure 7C,D). Immunofluorescence analysis for VCN shows that the protein is co-localized with actin in correspondence of focal adhesion formation on the substrate, and many focal adhesions were detectable in tenocytes cultured on either NC or MD-Tissue. Very frequently, the region corresponding to the presence of the focal adhesion seem bigger in tenocytes cultured on MD-Tissue compared to NC tenocytes (see arrows in Figure 7B). 

### 3.4. Wound Healing Assay

**Figure 8:**
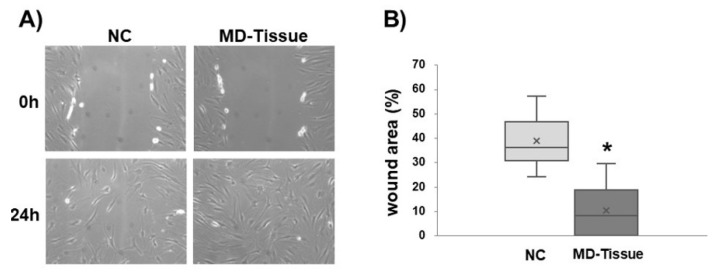
(**A**) Representative micrographs showing wound healing assay in control tenocytes (NC) and tenocytes grown on MD-Tissue at 0 and 24 h after the scratch. Original magnification: 10×. (**B**) Bar graphs showing the area of wound closure, expressed as a % of the area at 0 h, in cultured tenocytes in both experimental conditions 24 h after the scratch. * *p* < 0.005 vs. NC. ×: mean.

Wound healing assay was used to analyze tenocyte migration of tenocytes cultured on MD-Tissue. The comparison of the scratch size revealed that cell migration is significantly increased by MD-Tissue (*p* < 0.05 vs. NC) (Figure 8). 

## 4. Discussion

Treatment of tendinopathy, including Greater Trochanter Pain Syndrome and others, remain a big problem for clinicians, because its pathogenesis is still largely misunderstood, and many treatments have no real evidence. We aimed at characterizing in vitro the molecular mechanisms triggered in human tenocytes by MD-Tissue^®^, and the main findings of this study reveal that this collagen-based injectable medical compound could favor tendon repair by inducing tenocyte proliferation and migration, and stimulating COL-I synthesis, secretion and maturation. 

Tendons play key functions in musculoskeletal system serving to transfer forces generated by muscle contraction to the skeleton. Tendon mechanical properties are based on the underlying extracellular matrix (ECM) structure and composition, mainly consisting of type I collagen (COL-I), which constitutes about 60–85% of the dry mass of the tendon and about 95% of the total collagen, and elastin, accounting for 1–2% [13,14,15]. Collagen and elastin are embedded in a ground substance rich in proteoglycans, glycosaminoglycans, structural glycoproteins, and a wide variety of other small molecules, having water-binding capacity and contributing to the stabilization of the whole tendon structure. The unique structure and composition of tendons provide them the characteristic mechanical stability, and COL-I represents the most important factor for tendon mechanical strength.

Tenocytes are tendon specialized fibroblasts interspersed between collagen fiber bundles and aligned along the long axis of tendon, responsible for the synthesis and degradation of collagen and all components of the ECM. Tenocytes act as mechanosensors playing key roles for the tendon’s adaptive response and change their metabolic activities in response to mechanical forces acting on tendons, modifying gene expression for ECM components and, as a consequence, affecting tendon’s mechanical properties [14,16,17].

Tendon mechanical properties are based on its structure and composition, allowing tissue mechanical adaptation in response to mechanical forces. Tenocytes are responsible for tendon mechanical adaptation converting mechanical stimuli into biochemical signals that ultimately lead to tendon adaptive physiological or pathological changes. Mechanical loads at physiological levels are usually beneficial to tendons in terms of enhancing their mechanical properties [25,26,27], and increase collagen synthesis [28]. Therefore, appropriate mechanical loads induce tendon adaptation and have anabolic effects, improving their strength and healing quality after injury. 

Collagen is the major component of tendon ECM; its turnover is controlled by tenocytes acting at the level of collagen synthesis, maturation and degradation, thus determining the tendon ability to resist mechanical forces and repair in response to injury [14]. Our data show that COL-I secretion is significantly induced by MD-Tissue^®^ at all considered time points, suggesting that this medical compound is able to stimulate an anabolic phenotype of tenocytes. Newly synthesized collagen undergoes cross-linking, that is an important requirement for collagen maturation providing tendon strength, collagen fibril stabilization and increased tendon tensile strength [29,30]. Furthermore, our data show that this particular medical compound is not able to affect gene expression for LH2b, needed to provide cross-linking during collagen maturation, suggesting that collagen stability is not affected.

Since collagen content is the result of a finely regulated dynamic balance between its synthesis and degradation driven by MMPs, tendon strength is strongly dependent on degradation pathways. COL breakdown is played by MMP-1, able to cleave the intact collagen triple helix, followed by other proteases [31,32]. The role of MMP-1 is boosted by the previously demonstrated inverse correlation between MMP-1 gene/protein expression and the amplitude of tensile mechanical load on tendons, suggesting that low levels of MMP-1 lead to a more stable and less susceptible to damage tendon structure [33]. MMPs activation and activity are regulated by TIMPs [34,35], and TIMP-1 is the main inhibitor of MMP-1. In this study, we show that MMP-1 and MMP-2 were unaffected by MD-Tissue^®^, but, interestingly, we observed a significant increase in TIMP-1 gene expression induced by the medical compound after 24 h, and a tendency to an up-regulation after 72 h. This result leads to the hypothesis that MD-Tissue is able to stimulate COL secretion by tenocytes, increasing COL content in tendons, and that COL increase is favored by inhibition of its degradation. 

Some interindividual differences in fibroblast cell lines were observed, especially in LH2b mRNA, MMPs and TIMP-1 mRNA. In fact, in some tenocytes the expression resulted unchanged, increased or decreased. Interindividual differences were previously described in gingival fibroblasts, suggesting the “cell subpopulation theory” [36] that described that the gingival fibroblast population is composed of different subsets of fibroblasts that respond to external or endogenous stimuli in different way. Fibroblast heterogeneity was described also recently [37]. According to this theory, we can hypothesize it is valid also for fibroblasts derived from different tissues, such as tenocytes.

ECM homeostasis is influenced by mechanical forces acting on tendons, and tenocytes are key effectors in tendon ECM remodeling and adaptation to the experienced mechanical loading. They are mechanoresponsive cells able to convert mechanical signals into biological events such as gene expression and cell proliferation [38,39]. Tenocytes are able to sense changes in their mechanical environment using a mechanotransduction system based on the actin cytoskeleton, and to respond by modifying their activity [33]. Since actin cytoskeleton arrangement and integrity are the key mechanotransduction apparatus influencing the tensional homeostasis and mechanoresponsiveness needed to maintain ECM balance [40,41], we investigated whether MD-Tissue^®^ affected actin cytoskeleton. Our data show that actin filaments are brightly labelled and longitudinally running in tenocytes cultured on plastic (NC) or on MD-Tissue^®^, and their arrangement is similar in both experimental conditions, suggesting that this component of the mechanoresponsive apparatus is not modified by the medical compound. 

The actin cytoskeleton is also actively involved in cell migration, a dynamic process mediated by a repeated cycle of attachment to the ECM, generation of cytoskeletal forces of propulsion, and subsequent detachment of the cell from the matrix. The attachment of cells to the ECM is mediated by integrins, transmembrane proteins that provide a bridge though which forces can be transmitted between inside and outside of the cells. In fact, the extracellular domain of the integrin binds to ECM components, whereas its cytoplasmic domain links various intracellular proteins interposed between actin filaments and the integrin, forming focal adhesion complexes at the leading edge of the cell [42]. Focal adhesions are not only involved in transmitting the mechanical signals from the ECM into cells to modulate ECM remodeling; they are also necessary to generate the traction required for cell migration [42]. We used a wound healing assay to investigate if tenocyte motility is affected by MD-Tissue^®^ since this test is particularly suitable to investigate the effects of cell–matrix interactions on cell migration, mimicking in vivo cell migration [21], and we found that this medical compound significantly increased cell migration. Interestingly, no difference in actin cytoskeleton between NC and MD-Tissue tenocytes was detected, and also gene expression for FAK and VCN, two main components of the focal adhesion complex, was similar in both experimental conditions, supporting the hypothesis of a similar ability to form focal adhesions. However, interestingly, immunofluorescence analysis of VCN expression revealed that focal adhesions containing VCN seem more evident in tenocytes plated on MD-Tissue, leading to the hypothesis that MD-Tissue^®^ could act as a mechanical scaffold improving tenocyte focal adhesion, allowing a more efficient mechanoresponsiveness and migration ability. This could make tenocytes more efficient in maintaining ECM homeostasis in response to different mechanical load and in generating the traction necessary for cell migration [42]. Since the process of tendon repair and regeneration is reliant on tenocyte migration [43], MD-Tissue^®^ could be effective in favoring tendon healing in tendinopathies, but clinical trials are needed to confirm this suggestion. 

A limit of this study is represented by the lacking of studies aimed at characterizing the permanence and rheological and visco-elastic properties of MD-Tissue, however clinical studies are available demonstrating that the collagen-based medical compound is effective in osteoarticular pathologies [18,19]. 

MD-Tissue could offer some advantages compared to other biological agents since it can be administered singularly or in association with other therapeutic agents and the reduced cost compared to low or high MW HA could allow wider use, resulting in a NSAIDs intake reduction.

## 5. Conclusions

Considered as a whole, these in vitro findings suggest a mechanism for MD-Tissue likely acting as a mechanical scaffold able to induce an anabolic phenotype in tenocytes, favoring tendon homeostasis and repair. Interestingly, it was demonstrated [20] that the addition of acid-soluble type I collagen to fibroblasts cultured on plastic also affected collagen turnover mechanisms since some of the collagen comes out of solution and precipitates on cell surfaces, still eliciting some effect nonmediated by a cell-substrate interaction. This suggests that little mechanical resistance is sufficient to activate cell receptors, influencing collagen turnover mechanisms and, very likely, other biological activities played by tenocytes. We can hypothesize that MD-Tissue effect could be also mediated by this alternative mechanism. Our results suggest that MD-Tissue^®^ could represent a novel therapeutic approach, and stimulate new clinical trials to investigate the effects of this medical compound to treat difficult tendon pathologies. Based on our in vitro findings obtained on gluteal tenocytes, we will perform in future a clinical trial to prove its utility on GTPS. 

## Figures and Tables

**Table 1:** Eligibility Criteria.

Inclusion Criteria	Exclusion Criteria
Age ranging 18–70 years.	Patients diagnosed of great trochanter tendinopathy
Indication for total hip arthroplasty.	Patients affected by genetic collagen disorders.
Patients who signed written informed consent for the surgery.	Patients diagnosed of spondyloarthritis with involvement of the affected hip.
Patients able to understand the study conditions and willing to participate for its entire duration.	Patients affected by psoriatic arthritis.
	Drug-addicted Alcohol-addicted Psychiatric disorders Clinical conditions which could compromise the results of the surgical procedure or of the follow-up.
	Informed consent not accepted.
	Pregnant or breastfeeding women.
	Patients affected by diabetes mellitus.
	Patients who had taken fluoroquinolones within 30 days before the surgery.
